# Effect of gluteal muscle strengthening exercise on sagittal balance and muscle volume in adult spinal deformity following long-segment fixation surgery

**DOI:** 10.1038/s41598-022-13190-5

**Published:** 2022-05-31

**Authors:** Ki Young Lee, Jung-Hee Lee, Sang-Kyu Im

**Affiliations:** Department of Orthopedic Surgery, Graduate School, College of Medicine, Kyung Hee University, 23, Kyungheedae-ro, Dongdaemun-gu, Seoul, 130-872 Korea

**Keywords:** Outcomes research, Diseases, Health care

## Abstract

This study aimed to investigate the changes in gluteal muscle volume and the effects of such changes in spinal alignment as a result of postoperative gluteal muscle strengthening exercise (GMSE) in patients following long-segment fixation for adult spinal deformity (ASD). Eighty-three consecutive patients (average age, 70.1 years) were analyzed. Three-dimensional CT scans were conducted to obtain serial axial gluteus muscle image slices. The size of each muscle area in every image slice was measured by Computer Aided Design and the sum of each muscle area was calculated. At the last follow-up, the sagittal vertical axis was significantly greater in the basic postoperative exercise group (1.49 mm vs. 17.94 mm), and the percentage of optimal sagittal alignment was significantly higher in the GMSE group (97.8% vs. 84.2%). At the last follow-up, the gluteus maximus volume was significantly higher in the GMSE group (900,107.1 cm^3^ vs. 825,714.2 cm^3^, *p* = 0.036). For the increase in muscle volume after 1 year, gluteus maximus and medius volumes showed a significant intergroup difference (+ 6.8% vs. + 2.4% and + 6.9% vs. + 3.6%). The GMSE protocol developed in this study could effectively increase gluteal muscle volume and maintain the optimal sagittal balance in patients with ASD.

## Introduction

With the advancement of healthcare technology and a greater quality of life, the average human life expectancy has increased, and current trends indicate that society is progressively aging. With the pursuit of a more active senior lifestyle, there has been increasing research interest in the treatment of reduced musculoskeletal function due to aging and age-related disabilities caused by degenerative changes^1^. Adult spinal deformity (ASD) is a well-known age-related disability that has a serious impact on the physical health and quality of life of the patient as it causes sagittal malalignment arising from a wide spectrum of degenerative changes, including paraspinal muscle weakness^2^. Numerous studies have reported the effects of surgical treatment to improve pain and disability in patients with ASD, with a consistent focus on restoring and maintaining sagittal balance in the spine^3,4^.

However, clear guidelines are yet to be established for postoperative rehabilitation after deformity correction in patients with ASD, and there is currently no consensus regarding the timing and need for rehabilitation^5^. Spinal fusion with rigid instrumentation ensures adequate stability in the fusion area and promotes the mobility of the patient after surgery. Thus, the rehabilitation of most patients with spinal fusion begins with early mobilization, including sitting, standing, and walking with support^6^. Notably, early ambulation in elderly patients after spinal surgery is known to reduce the length of hospital stay and perioperative complications while enhancing functional outcomes^7^. Nevertheless, even if the basic principles and desired outcomes of postoperative rehabilitation for older patients are the same, it is not clear whether older patients with ASD can achieve similar successful outcomes. To design a suitable rehabilitation program for patients of relatively old age with ASD and degeneration or atrophy of the back muscle, particularly the lumbar extensor muscle, and with long-level fixation, it is necessary to recognize the limitations faced by these patients and set goals accordingly.

When performing deformity correction in patients with ASD, extensive dissection, retraction, denervation, and fusion for relatively long-segment fixation lead to changes in the structure and function of the paraspinal muscle^8^. There have also been reports of atrophy and changes in fatty infiltration of back muscles, including multifidus on MRI and CT of patients who underwent spinal fusion^9,10^. Yagi et al.^11^ reported that surgically treated patients with degenerative lumbar scoliosis showed degeneration of trunk muscles including psoas and multifidus rather than upper and lower extremity muscles. Therefore, we determined that there is a limit to paraspinal muscle strengthening exercises, including multifidus and erector spinae, in patients with ASD. In addition to the lumbar extensor muscle, we became interested in the gluteal muscle that uprights the trunk to maintain postural stabilization^12,13^. Considering the condition of elderly patients with ASD undergoing relatively long-segment fixation, we devised a novel gluteal muscle strengthening exercise (GMSE) for patients with ASD by modifying the previous gluteal muscle exercise.

One of the most significant characteristics of humans is bipedalism^14^. The gluteus muscle mediates the evolutionary postural change from quadrupeds to bipeds and uniquely distinguishes humans from other primates, playing a critical role in bipedalism^15^. This study aimed to investigate the changes in gluteal muscle volume and to determine the effects of such changes in gluteal muscle volume by postoperative GMSE on the maintenance and improvement of dynamic balance in spinal alignment by analyzing radiographic measures.

## Methods

### Patient selection

We performed a retrospective analysis of consecutive patients with ASD who were enrolled between 2016 and 2018. All procedures were indicated and performed in compliance with our department’s standards and the Declaration of Helsinki and every participant of this study provided written informed consent. This study was approved by the institutional review boards at Kyung Hee University hospital (KHUH 2020-10-009).

The inclusion criteria were as follows:

1. Patients aged ≥ 65 years with ASD accompanied by sagittal malalignment (sagittal vertical axis [SVA] greater than 50 mm, pelvic incidence [PI] minus lumbar lordosis [LL] greater than 10°, and pelvic tilt [PT] greater than 25°) with a minimum of 1-year follow-up after deformity correction.
2. Patients who clearly demonstrated atrophy of the back musculature on the cross-sectional area of a MRI and CT scan as a diagnostic criterion for lumber degenerative kyphosis (LDK) and clinical signs such as walking difficulty with stooping, inability to lift heavy objects to the front, difficulty in climbing slopes, and the need for elbow support when working in the kitchen, resulting in a hard corn on the extensor surface of the elbow^16,17^.
3. Patients who underwent long-segment fixation with sacropelvic fixation and setting the uppermost instrumented vertebra at the T10 level and the lowermost instrumented vertebra at the S1 level as a surgical treatment by a single surgeon at a single institution.

The exclusion criteria were as follows: (1) patients showing limited gait and reduced mobility after lower extremity surgery; (2) patients without the ability to stand independently or walk for normal exercise; and (3) patients with deformities resulting from trauma, spinal infection, ankylosing spondylitis, rheumatoid arthritis, neuromuscular disease, or tumors.

The patients who met the inclusion criteria in this study were randomly allocated to an exercise group that performed our novel GMSE after deformity correction from 2017 to 2018 and a control group that performed the basic postoperative exercise from 2016 to 2017.

### Radiographic measurement

Sagittal alignment was evaluated using lateral 14 × 36 inch full-spine radiographs obtained with the patient standing in a neutral, unsupported position with “fists-on-clavicle” position^18^. All preoperative, immediate postoperative, and 1-year postoperative digital radiographs were evaluated using validated software (Surgimap, Nemaris Inc, New York, NY, USA)^19^. All radiological parameters were measured by two professional orthopedic spine surgeons, and the mean measurements were used for the analysis.

We evaluated SVA, thoracic kyphosis (TK), LL, lumbosacral junctional angle (LS), PI, PT, and sacral slope (SS). PI, PT, SS, and postoperative PI-LL were measured using a standing lateral radiograph of the pelvis using methods described in previous reports^20,21^. Optimal and suboptimal sagittal alignment were defined as an SVA ≤ 50 mm and > 50 mm, respectively.

### Postoperative rehabilitation program

**Figure 1:**
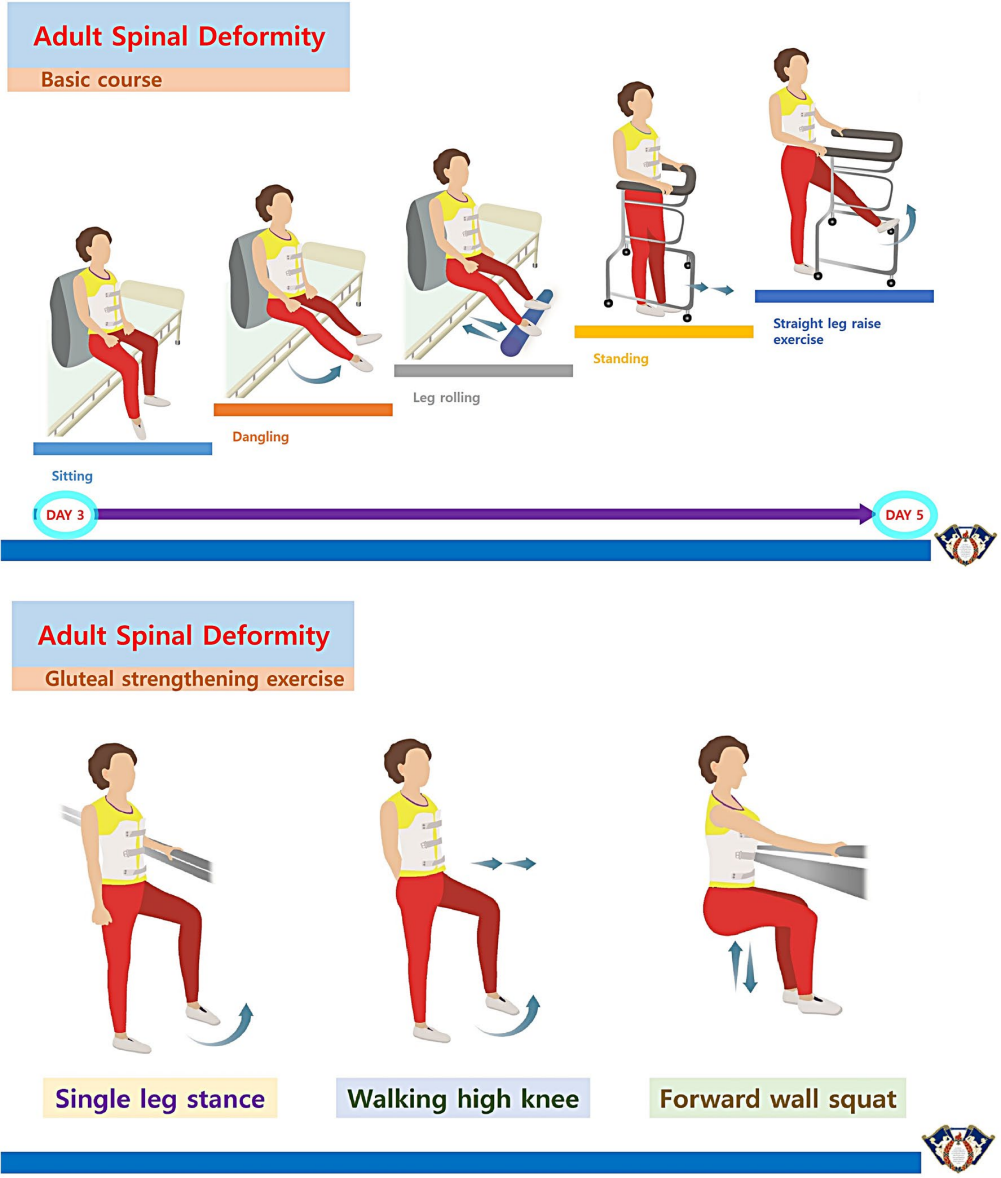
Schematics for our rehabilitation protocol for adult spinal deformity correction. The basic course consists of sitting, dangling, leg rolling exercises with sitting position, standing, and straight leg raising exercises. Gluteal muscle strengthening exercise consists of single leg stance exercise, walking high knee exercise, and forward wall squat exercise.

For all patients with ASD, the following postoperative rehabilitation protocol was used after deformity correction (Fig. 1). First, the patient is assisted to perform sitting, dangling, leg rolling exercises in the sitting position, standing, and straight leg raising exercises from the third day after surgery. Walking commences on the fifth day after surgery. Here, to prevent instrument failure or dislocation or breakage of pedicle screws, it is crucial to reduce the strain on the fused and adjacent segments, and with the start of walking, the lumbar spine should be maintained in the neutral position as much as possible during exercise^22^. Each exercise is performed for 20 min, three times a day, and was monitored during the length of stay and at every outpatient visit at 3-month intervals after surgery.

In addition to the basic postoperative rehabilitation protocol, patients were assisted to perform a gluteal muscle exercise to maintain postural stabilization through the upright trunk, commencing from the day ambulation was allowed following surgery (Fig. 1). Exercise status was monitored during the length of stay and at every outpatient visit at 3-month intervals after surgery. Various exercises such as single-limb squat, side-bridge, and forward step-up have been reported as exercises for strengthening the gluteus muscle^23^. However, it is crucial that the choice of exercise takes into account patient factors such as functional status, overall muscular strength, and postoperative health^23^. As patients with ASD generally show degeneration and atrophy of the paraspinal muscle, and as they undergo the long-level spinal construct and fusion, the gluteal muscle exercise is based on the following protocol that modifies known exercises, while maximally preserving the neutral position of the lumbar spine:

- *Single leg stance exercise* The patient holds the rail or the wall with one hand during exercise to maintain physical balance. The knee on one side with 90° flexion is lifted up to the hip joint position on the other side. The hip on the opposite side is maintained in extension to maximally preserve the upright position without swaying to the sides. This posture is maintained for 5 s, and the exercise is performed for 20 min, three times a day.
- *Walking high knee exercise* The patient places both hands in the waist area while maintaining an upright position. During a slow walk, force is applied to keep the knee on one side with 90° of flexion and the hip on the other limb in maximum extension. The exercise is performed for 20 min, three times a day.
- *Forward wall squat exercise* The patient faces the wall and holds the rail or edge of the desk with both hands. The starting point is standing with the knee and hip at 0° in the sagittal plane while the feet are positioned approximately 15° away from the midline to maintain the hip in a state of slight external rotation. The inter-pedal distance on the coronal plane is based on the shoulder width to maintain the hip in a state of slight abduction. Next, the knee joint is flexed at 90° while the patient applies force to the hip to stand up. The exercise is performed for 20 min, three times a day.

### Gluteus muscles volume measurement

**Figure 2:**
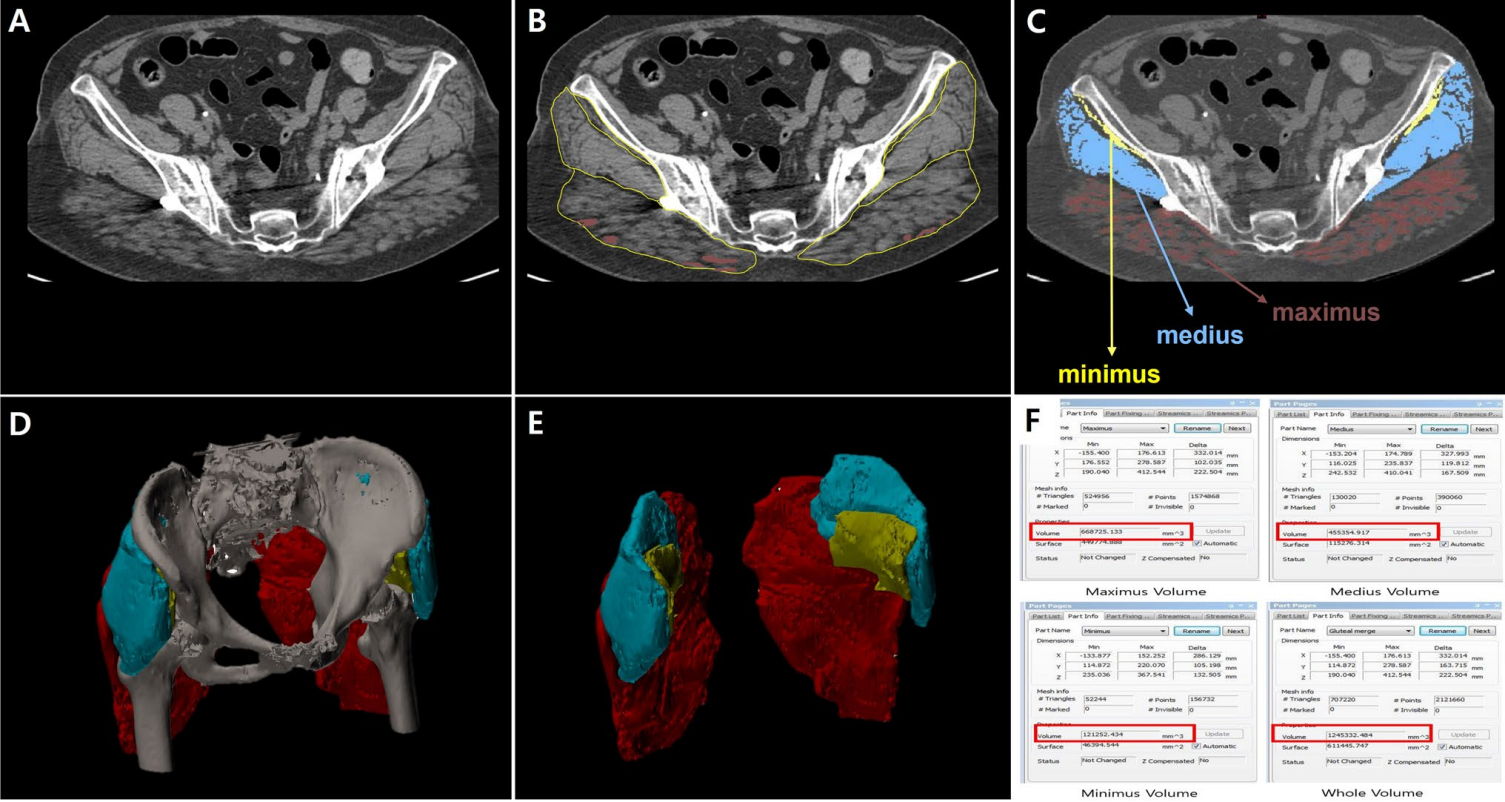
Gluteal muscle volume measurement; (**A**) A series of cross-sectional images of lumbosacral 3-dimentional CT. (**B** and **C**) After uploading to the computer aided design program, outlining and masking the gluteus maximus, medius, and minimus muscles. (**D** and **E**) After removing the fat tissue, abdominal organs and bony structures, obtaining the pure muscle mass. (**F**) Calculating the sum of the cross-sectional area of each muscle and estimating the volume of each muscle.

The gluteal muscle volume was measured using the lumbosacral three-dimensional (3D) CT (Ingenuity Core 128, Philips, Amsterdam, The Netherlands) taken immediately after surgery and one year after surgery (Fig. 2). A series of cross-sectional images from the iliac crest to the great tuberosity were obtained at 2.5 mm intervals, which included cross-sectional images of the gluteus muscles (Fig. 2A). These images were collected in the Digital Imaging and Communications in Medicine (DICOM) format, uploaded to the Computer Aided Design (CAD) program (Mimics 17.0, Materialize, Leuven, Belgium), and then applied to outline the gluteus maximus, medius, and minimus muscles (Fig. 2B and C). In addition, the CT attenuation threshold of 30–100 Hounsfield units (HU) was obtained, and after removing the fat tissue, abdominal organs, and bony structures (Fig. 2D and E), the size of the pure muscle mass was measured^24^. The sum of the cross-sectional area of each muscle was calculated, which was multiplied by the thickness (2.5 mm) to estimate the volume of each muscle (Fig. 2F)^25^, and the percent change in volume between the postoperative period and last follow-up was calculated. Measurements were taken twice by each of two orthopedic surgeons with at least two years of fellowship training in spinal surgery, and the mean was calculated for subsequent analyses.

### Clinical outcome assessment

Clinical assessment was performed using the Oswestry Disability Index (ODI) and visual analog scale (VAS) for back pain and radiating pain. The preoperative and last follow-up values of the two groups were compared.

### Statistical analysis

For continuous variables, analysis of variance with an unpaired t-test was used for variables with normality, and the Wilcoxon's rank-sum test was used for variables without normality. Categorical variables were assessed using the chi-square test and compensated using Fisher’s exact test to identify the association between GMSE and optimal balance maintenance. The interclass correlation coefficient (ICC) was estimated to assess the intra- and interobserver reliability of the gluteal muscle volume measurements by the two orthopedic surgeons. An ICC of 0.75 was set as the reliability threshold^26^. All statistical calculations were performed using SPSS software (version 25.0. IBM Corp., Armonk, NY, USA). Statistical significance was set at *p* < 0.05.

## Results

### Baseline characteristics

Table 1 presents the baseline characteristics of the patients. At the time of the study, the database included 104 surgical patients. Based on the inclusion and exclusion criteria, 83 patients were selected for analysis. The patients consisted of 83 women, and the average age at surgery was 70.1 years. All patients received sacropelvic fixation to increase the stability of the sacropelvic area. Baseline characteristics were not significantly difference between the two groups (*p* > 0.05).

**Table 1:** Demographics and baseline data.

Variable	Exercise group (n = 45)	Control group (n = 38)
**Gender**		
Male	0	0
Female	45	38
Age at operation (years)	68.93 ± 6.203	70.18 ± 5.844
BMD (g/cm^2^)	0.9758 ± 0.21127	0.9690 ± 0.17196
BMI (kg/m^2^)	25.2 ± 3.3	25.1 ± 3.2
Follow-up (months)	12	
UIV	T10	
LIV	S1	
Fused segments	8	
Sacropelvic fixation	45	38

Data are presented as mean ± standard deviation or number.

*BMD* bone mineral density, *BMI* body mass index, *UIV* uppermost instrumented vertebra, *LIV* lowermost instrumented vertebra.

### Comparison of radiographic parameters between the two groups

Table 2 shows a comparison of the radiographic parameters between the two groups. SVA was 199.8 mm in the exercise group and 199.9 mm in the control group before surgery and improved to − 8.6 mm and − 17.2 mm, respectively, after surgery; there was no significant difference between the groups. The last follow-up SVA was 1.49 mm and 17.94 mm, respectively, indicating a significantly greater SVA in the control group (*p* = 0.016). In addition, compared to the immediate postoperative period, the control group showed a greater margin of increase in SVA (+ 35.12 mm in the control group vs. + 10.05 mm in the exercise group, *p* = 0.002).

**Table 2:** Comparison of radiographic parameters and clinical outcomes.

Variables	Exercise group (n = 45)	Control group (n = 38)	*P* value
**Sagittal vertical axis (mm)**			
Preoperation	199.78 ± 84.257	199.93 ± 57.811	0.992
Postoperation	− 8.56 ± 31.894	− 17.18 ± 26.973	0.192
Last follow-up	1.49 ± 27.742	17.94 ± 33.042	0.016*
Change	10.05 ± 26.679	35.12 ± 40.849	0.002*
**Thoracic kyphosis (°)**			
Preoperation	5.35 ± 15.456	2.97 ± 15.746	0.489
Postoperation	28.15 ± 15.534	24.25 ± 10.583	0.181
Last follow-up	35.86 ± 17.749	31.01 ± 15.318	0.184
Change	7.72 ± 8.006	6.75 ± 7.305	0.567
**Lumbar lordosis (°)**			
Preoperation	0.92 ± 18.305	6.36 ± 19.015	0.142
Postoperation	− 65.82 ± 14.263	− 65.05 ± 13.222	0.799
Last follow-up	− 65.00 ± 16.624	− 55.94 ± 32.033	0.102
Change	0.82 ± 7.529	9.11 ± 31.036	0.087
**Lumbosacral junction angle (°)**			
Preoperation	− 6.4 ± 15.249	− 5.11 ± 16.501	0.713
Postoperation	− 27.45 ± 8.39	− 28.94 ± 8.524	0.427
Last follow-up	− 27.13 ± 10.165	− 28.53 ± 9.633	0.525
Change	0.32 ± 6.778	0.41 ± 6.166	0.951
Pelvic incidence (°)	54.59 ± 9.869	52.09 ± 12.029	0.192
**Sacral slope (°)**			
Preoperation	24.14 ± 10.95	21.82 ± 12.334	0.365
Postoperation	45.58 ± 9.193	43.17 ± 8.952	0.233
Last follow-up	43.37 ± 9.9	41.92 ± 7.648	0.463
Change	− 2.21 ± 7.132	− 1.25 ± 5.97	0.515
**Pelvic tilt (°)**			
Preoperation	31.44 ± 10.97	31.28 ± 15.379	0.956
Postoperation	10.01 ± 13.195	9.92 ± 14.631	0.977
Last follow-up	12.22 ± 13.538	11.18 ± 13.194	0.725
Change	2.21 ± 7.132	1.25 ± 5.97	0.515
Pelvic incidence–postoperative lumbar lordosis	− 11.23 ± 17.623	− 12.96 ± 17.193	0.656
**VAS (back pain)**			
Preoperation	7.7 ± 1	7.3 ± 1.6	0.221
Last follow-up	2 ± 1.3	2.5 ± 1.6	0.135
**VAS (radiating pain)**			
Preoperation	6.6 ± 1.8	6.1 ± 2.2	0.251
Last follow-up	2.5 ± 1.4	2.1 ± 1.3	0.217
**ODI**			
Preoperation	33.4 ± 5.6	32 ± 4.3	0.216
Last follow-up	13.8 ± 5.9	14.8 ± 5.9	0.444

Data are presented as mean ± standard deviation.

*p < 0.05.

*VAS* visual analog scale, *ODI* Oswestry disability index.

TK, LL, and LS improved in both groups, with no significant differences between the groups (*p* > 0.05). Postoperative PI-LL was − 11.23 and − 12.96, respectively, without significant difference between the two groups. Both groups showed favorable outcomes in terms of the pelvic parameters without significant differences (*p* > 0.05).

### Comparison of achievement and maintenance of optimal sagittal alignment between the two groups

In the immediate postoperative period, the achievement of optimal sagittal alignment was observed in both the exercise and control groups (Table 3). However, at the last follow-up, the percentage of optimal sagittal alignment in the exercise group was 97.8% (44/45), which was significantly higher than that in the control group (84.2% [32/38]) (*p* = 0.044).

**Table 3:** Achievement and maintain of optimal sagittal alignment between two groups.

Variables	Exercise group (n = 45)	Control group (n = 38)	*P* value
Immediate postoperative optimal alignment achievement	45 (100%)	38 (100%)	–
Last follow-up optimal alignment maintenance	44 (97.8%)	32 (84.2%)	0.044*

*p < 0.05.

### Comparison of gluteal muscle volumes between the two groups

In the immediate postoperative period, the gluteus maximus, medius, and minimus volumes did not significantly differ between the exercise and control groups (Table 4). At the last follow-up, the gluteus medius and minimus volumes showed no significant intergroup difference; the gluteus maximus volume was significantly higher in the exercise group (900,107.1 cm^3^ vs. 825,714.2 cm^3^, *p* = 0.036). For the increase in muscle volume after one year, the gluteus maximus and medius volumes showed a significant intergroup difference (+ 6.8% vs. + 2.4%, *p* < 0.001 and + 6.9% vs. + 3.6%, *p* = 0.024). ICCs (intra- and interobserver reliability) of both groups were high (0.86–0.94 and 0.83–0.92, respectively), indicating that the measures were reliable.

**Table 4:** Comparison of gluteal muscle volume between two groups.

Variables	Exercise group (n = 45)	Control group (n = 38)	*P* value
**Gluteus maximus volume (cm**^**3**^**)**			
Postoperation	842,032.7 ± 131,576.40	807,391.2 ± 160,732.79	0.283
Last follow-up	900,107.1 ± 155,771.81	825,714.2 ± 162,126.2	0.036*
Volume change (%)	6.8 ± 6.81	2.4 ± 3.82	< 0.001*
**Gluteus medius volume (cm**^**3**^**)**			
Postoperation	434,265.7 ± 69,963.51	435,259.9 ± 84,151.66	0.953
Last follow-up	461,477.4 ± 64,604.78	450,723.4 ± 88,250.89	0.524
Volume change (%)	6.9 ± 7.80	3.6 ± 5.17	0.024*
**Gluteus minimus volume (cm**^**3**^**)**			
Postoperation	93,150.5 ± 32,583.06	102,674.4 ± 28,316.65	0.163
Last follow-up	99,051.4 ± 31,366.92	107,978.8 ± 31,667.90	0.202
Volume change (%)	8.5 ± 16.40	5.1 ± 11.17	0.284

Data are presented as mean ± standard deviation.

*p < 0.05.

### Clinical outcomes

In both groups, VAS of back pain and radiating pain, and ODI, were improved at the last follow-up compared to before surgery without significant difference (Table 2).

## Discussion

This study was motivated by the need to find ways to maintain the optimal sagittal balance in patients who underwent long-segment fixation surgery for ASD. The premise was that GMSE, among various postoperative physical exercises, would increase the gluteal muscle volume and assist in maintaining the restored sagittal balance. The quality of postoperative rehabilitation has a significant influence on reducing subsequent disability, recurring injury, and the additional use of health care services^27^. While there have been significant advances in deformity correction for patients with ASD with an increase in the number of surgical procedures over the past decades, the optimal rehabilitation program during the postoperative period remains unclear. The findings of this study suggest an effective exercise protocol for patients after surgical treatment for ASD, contributing to the improvement in surgical outcomes.

### Gluteal muscle and rehabilitation

Humans are the only vertebrates that maintain an upright, totally vertical, bipedal position^14^, and the gluteus muscle plays a critical role in bipedalism^28^. The gluteus maximus muscle, in particular, is known as the significant muscle that allows numerous daily activities as well as the maintenance of the upright position^28,29^. Thus, the strengthening exercise of the gluteus muscle was predicted to assist in maintaining sagittal balance and preventing sagittal decompensation after ASD surgery. Many studies have reported the importance of rehabilitation of the gluteus muscle, but the main focus of these studies has been on lower extremity disorders^15^, and there have been no guidelines describing the need for, or recommended content of postoperative gluteal muscle rehabilitation after spinal surgeries, including deformity correction in ASD.

The known strengthening exercises for the gluteus muscle include lunge, bridging, squats, deadlifts, clams, leg presses, and step-ups. Reiman et al.^23^ reported that, during rehabilitative exercises, a very high-level activation on the electromyography (EMG) was observed for the gluteus maximus muscle in the forward step-up and for the gluteus medius muscle in single-limb squat and side-bridge to neutral spine position. Neto et al.^30^ also found that the step-up exercise and its variations presented the highest levels of gluteus maximus muscle activation. However, these studies were limited to healthy individuals, and their application to elderly patients with ASD following long-segment fixation from T10 to S1, with degeneration and atrophy in the paraspinal muscle, remains unclear. This study developed a GMSE protocol for elderly patients with ASD through the modification of the forward step-up and squat exercises that induced a high-level activation of the gluteus muscle in the two aforementioned studies.

Step-up exercise is related to the stabilization of the hip and knee in the upward and downward movement, while it extends the hip joint and maintains the pelvis level controlling excessive femur adduction and medial rotation^30^. Through the modification of the step-up exercise, the single leg stance exercise and the walking high knee exercise were applied to patients after ASD surgery. In the single-leg stance exercise, the patient is instructed to move as if to climb a stair from a static posture by lifting the knee on one side at 90° flexion to the position of the hip joint on the other side while maintaining the hip extension on the opposite side to maximally preserve the upright position and avoid swaying to the sides. In the walking high knee exercise, the single-leg stance exercise is maintained with the addition of a dynamic component in which the patient is guided to perform a slow walk. Both exercises were designed to determine the effects of the step-up exercise with maximal preservation of the neutral position.

A squat is a complex movement exercise involving the ankle, knee, and hip joints. The gluteus maximus muscle is activated throughout the squat exercise, and notably, the highest activation was reported based on EMG analysis for 90–60° hip flexion in the ascent phase^31^, while another study reported a higher EMG activity of the gluteus medius muscle during the squat exercise including wall squat and mini squat compared to other exercises^32^. Thus, noting the importance of squats in the GMSE, a forward wall squat exercise was developed for patients with ASD who are older and lack strength in the lower extremity. In the forward wall squat exercise, the patient is guided to hold the rail or the edge of the desk with both hands while maximally preserving the neutral position of the spine. The feet are positioned approximately 15° towards the exterior from the midline so as to maintain the hip in a state of slight external rotation. The inter-pedal distance on the coronal plane is based on the shoulder width to maintain the hip in a state of slight abduction. Next, the knee joint is flexed at 90°, while the patient applies force to the hip to stand up. McCaw et al.^33^ reported that, compared to the narrow stance, the wide stance of 140% shoulder width resulted in higher EMG activity of the gluteus maximus muscle. Thus, considering the physical state of patients with ASD, the exercise involved separating the feet distanced based on shoulder width.

### Effect of gluteal muscle strengthening exercise and ASD

The radiographic measurements showed that, in both groups, radiographic improvements in SVA, PT, and TK were observed following lumbar lordosis correction and at the last follow-up, and at the one-year follow-up, the optimal sagittal alignment was seen to have been maintained in 91.5% of patients. However, the percentage of patients showing the optimal sagittal alignment in the exercise group was 97.8%, which was significantly higher than that in the control group (84.2%), while a significantly higher value was observed for the last follow-up SVA and SVA change in the control group. These results suggest that GMSE in combination with the basic postoperative rehabilitation protocol could be a method to prevent sagittal decompensation in patients with ASD after surgery. To corroborate the results in this study, the actual margin of increase in the gluteus muscle after the postoperative GMSE was quantitatively evaluated by measuring the gluteal muscle volume using CAD and 3D-CT data obtained immediately after surgery and at one year after surgery.

First, the 3D-CT data were applied to the CAD program, where the fat tissue was removed through the HU border settings to estimate the area of the pure muscle mass. Next, by estimating the area of the overall cross-sectional images rather than the area of a single image based on the reference point, the errors from the cutting position in a single image were minimized, and the volume that approximated the actual whole muscle was obtained. Based on the results of these accurate and reliable analyses, a significantly higher gluteus maximus volume was observed in the exercise group than in the control group at the one-year follow-up, while significantly higher gluteus maximus and medius volumes were observed at the one-year follow-up.

**Figure 3:**
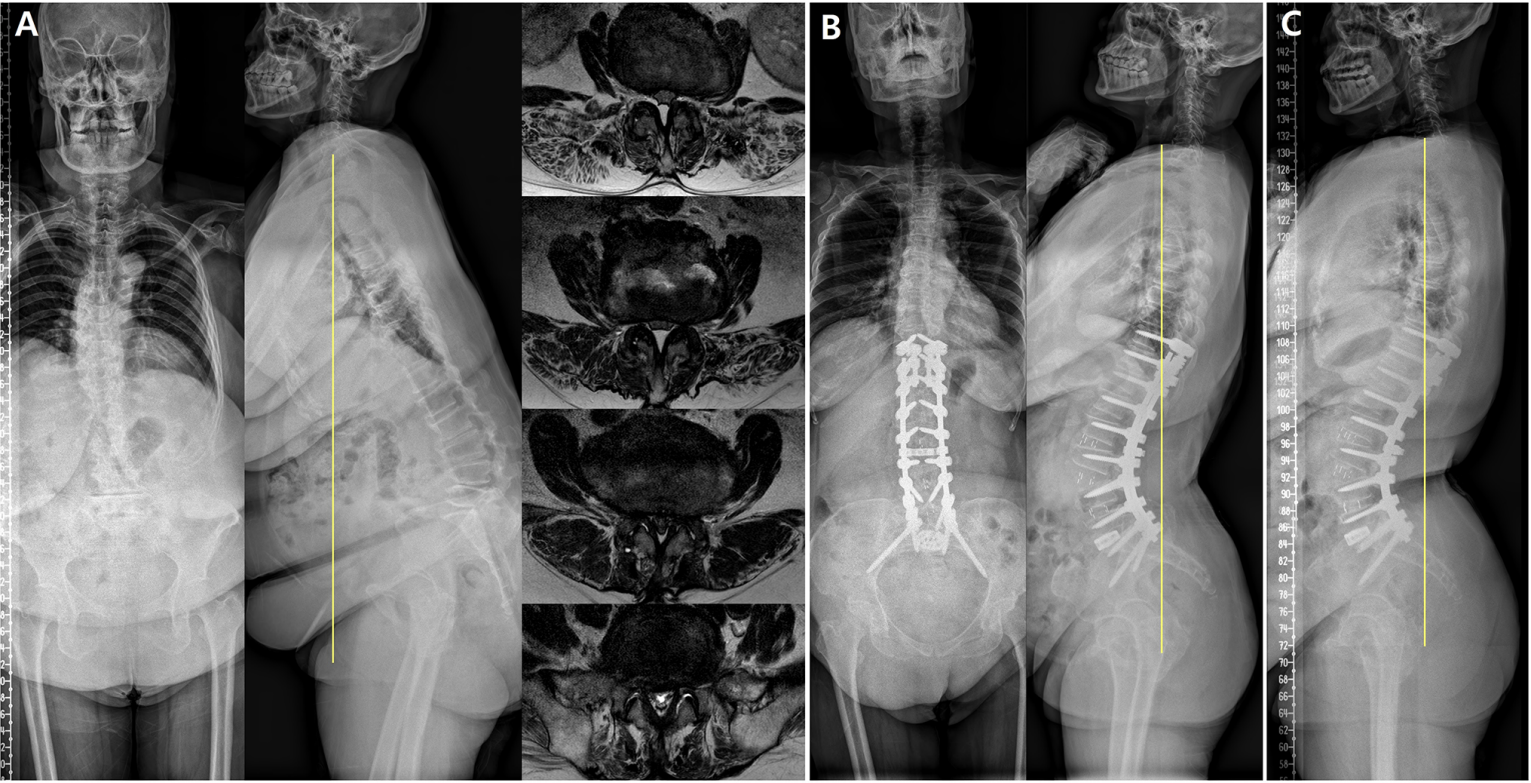
Radiographs showing a 70-year-old female with (**A**) degenerative sagittal imbalance (sagittal vertical axis 167 mm, pelvic incidence 40°, pelvic tilt 31°) who clearly showed atrophy of the back musculature on the cross-sectional area of MRI. (**B**) Oblique lumbar interbody fusion on L2-5, anterior lumbar interbody fusion on L5-S1, and posterior spinal fusion from T10 to S1 was performed (sagittal vertical axis − 60.9 mm, pelvic tilt 6.8°, whole gluteal muscle 1015.5 mm^3^). (**C**) After performing our basic and gluteal muscle strengthening exercise protocol during 1 year, the radiograph showed a well-maintained optimal sagittal balance and an increased shading of the gluteal muscle (sagittal vertical axis − 54.6 mm, pelvic tilt 12.6°, whole gluteus muscle 1132.7 mm^3^).

For patients with ASD, restoring sagittal balance is an important surgical goal, and its maintenance is also crucial^3^. However, pain levels may increase again with postoperative sagittal decompensation and mechanical complications such as pseudarthrosis and proximal junctional kyphosis^3,34,35^. Kim et al.^35^ reported that sagittal decompensation was observed in 23% of patients after ASD surgery, and extensor muscle weakness due to aging was identified as one of various causal factors. Inami et al.^34^ also reported that sagittal decompensation was observed in 15.4% of patients after ASD surgery, and a probable cause was inadequate muscle strength due to aging. Notably, we included and analyzed patients with a single etiology among those with ASD, namely LDK^16^. In patients with LDK, the paraspinal muscles, particularly the multifidus and erector spinae muscles, tend to decrease in mass to a greater extent as patients grow older^36,37^. Thus, for patients with ASD showing paraspinal muscle weakness and who are older, the gluteal muscle exercise protocol developed in this study is an adequately suitable exercise protocol after deformity correction surgery. It is anticipated that this protocol will improve surgical outcomes (Fig. 3).

### Limitations

This study had a few limitations. First, the one-year follow-up period was inadequate. Further studies should be performed with a longer follow-up period. Second, there may be problems with post-discharge adherence to rehabilitation programs, including GMSE. However, the deformity correction for patients with ASD at our hospital is performed for primarily for those patients who have a high desire for recovery to a normal life due to the deterioration of their basic life quality. We tried to increase adherence to rehabilitation by providing information and education to patients and their family members before surgery, and monitoring the exercise status at every outpatient visit at short intervals (3-month) after surgery. Using this approach, we were able to get satisfactory results. Third, the level of gluteal muscle activation as a result of exercise in this study was not identified. This should be addressed in future studies using an electromyogram. Despite these limitations, the significance of this study lies in its being the first to analyze the effects of a suitable exercise for patients after ASD surgery. Importantly, this study evaluated the positive effects of our novel protocol of GMSE and all muscle volume measurements were performed using a series of cross-sectional images rather than a single image. Furthermore, this study was able to produce reliable results by increasing the accuracy of gluteal muscle volume measurement using CAD.

## Conclusions

Based on the findings of this study, the GMSE protocol developed in this study could effectively increase the gluteal muscle volume in patients undergoing long-segment fixation for ASD. The protocol was also effective in maintaining the optimal sagittal alignment after surgery. Thus, our novel GMSE protocol will contribute to improving the surgical and functional outcomes of patients with ASD and will serve as a useful guideline for spinal reconstruction surgery in patients with ASD.

## Data Availability

All data analyzed during this study will be made available by the corresponding author on reasonable request.
